# Liquid biopsy, using a novel DNA methylation signature, distinguishes pancreatic adenocarcinoma from benign pancreatic disease

**DOI:** 10.1186/s13148-022-01246-2

**Published:** 2022-02-22

**Authors:** Lukas Vrba, Bernard W. Futscher, Marc Oshiro, George S. Watts, Emmanuel Menashi, Charles Hu, Hytham Hammad, Daniel R. Pennington, Umamaheshwari Golconda, Hemanth Gavini, Denise J. Roe, Rachna T. Shroff, Mark A. Nelson

**Affiliations:** 1 The University of Arizona Cancer Center, Tucson, AZ USA; 2 Department of Pathology, College of Medicine, University of Arizona, Tucson, AZ USA; 3 Honor Health Hospital, Scottsdale, AZ USA; 4 Dignity Health Chandler Regional Medical Center, Chandler, AZ USA; 5 Division of Hematology/Oncology, Department of Medicine, University of Arizona Caner Center, Tucson, AZ USA; 6 Department of Pharmacology and Toxicology, College of Pharmacy, University of Arizona, Tucson, AZ USA; 7 Department of Medical Pharmacology, College of Medicine, University of Arizona, Tucson, AZ USA; 8 Precision Epigenomics Inc, Tucson, AZ USA

## Abstract

We tested the ability of a novel DNA methylation biomarker set to distinguish metastatic pancreatic cancer cases from benign pancreatic cyst patients and to monitor tumor dynamics using quantitative DNA methylation analysis of cell-free DNA (cfDNA) from blood samples. The biomarkers were able to distinguish malignant cases from benign disease with high sensitivity and specificity (AUC = 0.999). Furthermore, the biomarkers detected a consistent decline in tumor-derived cfDNA in samples from patients undergoing chemotherapy. The study indicates that our liquid biopsy assay could be useful for management of pancreatic cancer patients.

**Supplementary Information:**

The online version contains supplementary material available at 10.1186/s13148-022-01246-2.

## Introduction

Pancreatic adenocarcinoma (PDAC) is a relatively rare disease (3% of all cancer cases) [1]; however, due to mostly late stage diagnosis, it has a 5-year survival rate of only 10% [2] and is the third leading cause of cancer death in the USA [1]. Earlier diagnosis of cancer or its recurrence [3] may allow earlier intervention and could improve management of the disease [4]. Clinically available blood biomarkers, such as carbohydrate antigen 19–9, are unreliable for detection early-stage pancreatic cancer, can give false positive results in the setting of inflammatory pancreatitis and biliary obstruction, and may be normal in advanced disease subjects who do not express Lewis blood group antigens [5, 6].

One means to improve early pancreatic cancer detection and monitor disease burden during treatment could be a liquid biopsy approach. A liquid biopsy involves examining cancer-related material (i.e., DNA) from a blood sample. The liquid biopsy techniques are based on detecting tumor-specific biomarkers in cell-free DNA (cfDNA) fraction of blood samples in which the circulating tumor DNA (ctDNA) resides [7–11]; the ctDNA fraction varies based on tumor type and disease progression [12–14]. Methylated DNA markers appear to be more broadly informative than DNA mutations [15]. In addition, DNA methylation could be specific to tumors arising in different organs and tissues [11]. Since tumors have many aberrantly methylated DNA regions [16–18], multiple genomic loci could be analyzed using conventional methods like DNA methylation-specific qPCR [19] for the presence of tumor-specific DNA methylation; that increases the sensitivity of the technique. Thus, the detection of tumor-specific DNA methylation in cfDNA from blood liquid biopsies could be used for diagnosis and monitoring of pancreatic cancer.

We previously reported in silico identification of a large suite of DNA methylation loci specifically hypermethylated in common human cancers that could be used as epigenetic biomarkers [20]. Testing of these markers on independent GEO data, including data from a different analytical platform, confirmed their ability to distinguish tumors from normal tissues with high sensitivity and specificity [20]. In addition, we have shown that the DNA methylation biomarker loci acquire aberrant methylation early in cancer progression and have thus potential to detect early stages of the disease [21]. We further selected a set of 10 DNA methylation biomarkers that detect common carcinomas including PDAC, with high sensitivity and specificity [22]. Using independent epigenomic data from the Gene Expression Omnibus (GEO) database, we demonstrated that this 10 marker set could identify, with high sensitivity and specificity, all carcinoma types it was designed for (i.e., bladder, breast, colorectal, esophageal, oral, non-small cell lung (NSCLC), pancreatic, and prostate cancer) [22]. Clinical testing using DNA methylation-specific qPCR analysis of patient plasma samples showed that this 10 marker set could distinguish NSCLC cases from controls with high sensitivity and specificity (AUC = 0.956), and furthermore, the signal from the markers correlates with tumor size and decreases after surgical resection of lung tumors [22]. We have also shown that the signal from the biomarkers does not depend on sex and only slightly increases with the age of the subjects [22].

The purpose of the current study was to test our DNA methylation biomarker set on blood samples from metastatic pancreatic cancer patients and patients with benign pancreatic cysts disease. The DNA methylation signal from the markers was able to distinguish between patients with malignant disease and those with benign pancreatic cysts with high sensitivity and specificity (AUC = 0.999). We demonstrate here that the biomarker set can detect pancreatic cancer in the plasma of metastatic patients and can distinguish malignant pancreatic cancer from benign pancreatic cysts.

## Methods

### Participants

The studied population (Table 1) consisted of pancreatic cancer patients and patients with benign pancreatic disease recruited between 2017 and 2020 at the University of Arizona Cancer Center, Tucson, Arizona, USA, and HonorHealth Hospital, Scottsdale, Arizona, USA). Institutional Review Board approval from both institutions was obtained prior to the study initiation and all participants provided written informed consent. The cancer cohort consisted of 19 metastatic pancreatic ductal adenocarcinoma cases; the patients did not undergo to surgery resection of the tumor due to metastatic disease. A cohort of 44 patients with benign pancreatic cysts was used as a control. In addition, a cohort of 9 patients undergoing experimental treatment of metastatic pancreatic adenocarcinoma had samples collected before (cycle C1) and 4 weeks after the start of treatment (cycle C2). All cancer cases had pathologically confirmed pancreatic cancer at the time of blood draw.

**Table 1:** Participant demographic information

Characteristic	Malignant (n = 19)			Benign (n = 44)			Treated (n = 9)		
	No		%	No		%	No		%
Age, years									
Median		67			75			71	
Range		47–80			39–93			60–75	
Sex									
Male	17		89	20		45	6		67
Female	2		11	24		55	3		33
Disease Type									
Pancreatic Ductal Adenocarcinoma (stage IV)	19		100	–		–	9		100
Benign Pancreatic Cyst	–		–	44		100	–		–
CA 19–9 (U/mL)									
Median		1173			NA			1769	
Range		3–56,534			NA			14–72,745	
Tumor Burden (cm)									
Median		10.5			–			NA	
Range		0–27.4			–			NA	
Number of Metastatic Sites									
Median		2			–			NA	
Range		1–5			–			NA	
Number of Lymph Nodes									
Median		2			–			NA	
Range		0–7			–			NA	

### Blood sample processing and two-step qPCR

Whole blood was collected in Streck cell-free DNA BCT tubes (La Vista, NE) and stored for no longer than 3 days at room temperature before further processing. The blood plasma collection and storage, the cfDNA extraction and storage, and the cfDNA sodium bisulfite treatment were performed as described earlier [22]. The set of ten biomarkers that identifies most tumors of common carcinoma types (Additional file 1: Table S1**)** and the design of qPCR amplicons specific for the biomarker loci were described before [20, 22]. The two-step qPCR consisting of 15 cycles of pre-amplification using cocktail of all primer pairs and 50 cycles of loci-specific qPCR detection was done as previously described by our group [22].

### qPCR data analysis

The threshold cycles (Cts) for individual amplicons were determined using fixed marker-specific thresholds to keep consistency between individual qPCR runs. Undetermined Cts or Cts higher than 40 were set to 40. The data were then converted by a formula 40-Ct. This way Ct 40 was set as a background (zero) and the values that are still in log2 transformed scale but are increasing with the level of DNA methylation-specific signal were obtained. These values for all markers or the means of these values for all markers were used in the plots and ROC analysis. The ROC analysis and AUC calculations were performed using the R library pROC [23], HRs and KM plots were computed using the R library survival [24]. Data were normalized to 2 ml of plasma and data from longitudinal samples was further normalized for cfDNA load using the mean of the three universally methylated control amplicons [22]. Since the DNA methylation signal from the biomarkers spans several orders of magnitude, nonparametric tests were used to test differences between the groups (Wilcoxon rank sum test or Wilcoxon signed rank test for dependent longitudinal samples). Normal human blood DNA (20 ng, 1:1 mix of male and female, Promega, G147A, G152A) spiked with 1% of DNA from MDA-MB231 cancer cell line, that has all marker loci fully methylated, was used as a positive control.

## Results

**Fig. 1:**
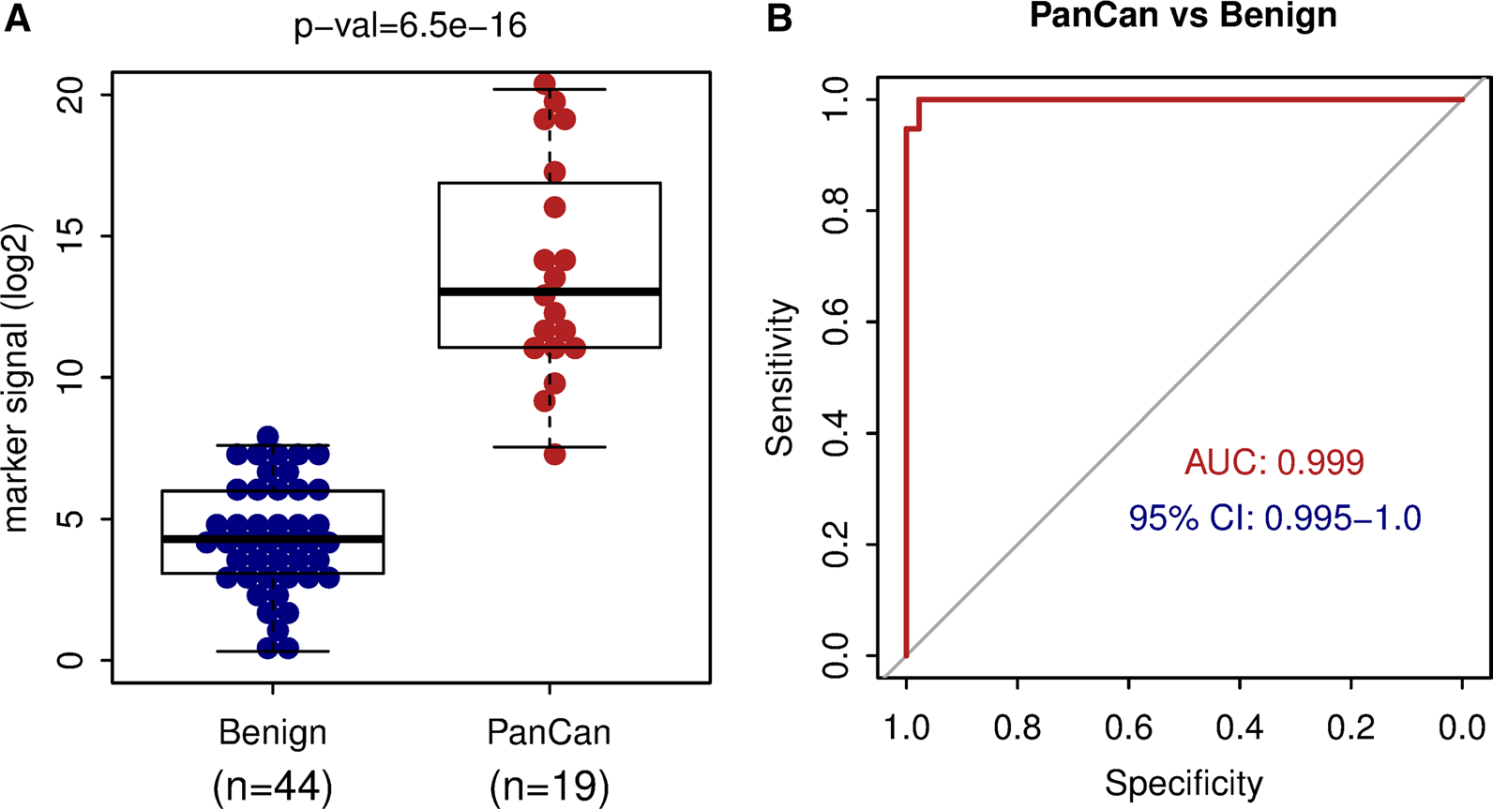
The DNA methylation biomarker set differentiates between pancreatic adenocarcinoma and benign pancreatic disease with high sensitivity and specificity. **A** Mean DNA methylation signal per marker for the control group of 44 patients with benign pancreatic disease and for the group of 19 pancreatic ductal adenocarcinoma cases. The y-axis is in a log2 scale. *p* = 6.5 × 10^–16^ by Wilcoxon rank sum test. **B** The receiver operating characteristic (ROC) analysis of the biomarker set signal from 44 controls and 19 cancer cases. AUC area under the curve, CI confidence interval

To test the ability of the biomarkers to detect pancreatic cancer, we analyzed DNA methylation levels of the biomarkers in cfDNA from pancreatic cancer patients and patients with benign pancreatic disease. The cfDNA was extracted from plasma samples of 44 patients with benign pancreatic cysts (benign group) and 19 pancreatic adenocarcinoma patients (malignant group) (Table 1). While cfDNA from patients with benign disease shows relatively low background DNA methylation across the biomarker set, the pancreatic adenocarcinoma patient samples displayed a markedly higher level of DNA methylation signal, and many of the cancer patients show high-level DNA methylation across most of the biomarkers (Fig. 1, Additional file 1: Figure S1). All (19 of 19) cancer patients (100%) have DNA methylation signal higher than the 95th percentile of the benign group (Additional file 1: Figure S1); this suggests sensitivity 100% at 95% specificity. The distribution of the mean DNA methylation signal from all biomarkers in the malignant group is highly significantly different (*p* value = 6.5 × 10^–16^) from the signal in the benign group (Fig. 1A). The median DNA methylation of the biomarker set is 426-fold higher in the malignant than in the benign group (Fig. 1A). The ROC analysis using data from the 44 subjects from control benign group and 19 subjects with malignant disease revealed large area under the curve (AUC = 0.999) with 95% confidence interval 0.995–1.0 (Fig. 1B). Furthermore, the biomarker DNA methylation signal was significantly increased in patients with liver metastasis and was able to distinguish them from PDAC patients without liver metastasis (AUC = 0.91, Additional file 1: Figure S2). Since carbohydrate antigen 19–9 (CA 19-9) levels at baseline and a follow-up were available for the malignant cases, we tested how the signal from the biomarkers compares to CA 19-9 as a potential predictor. While CA 19-9 had no prognostic value in the studied cohort (HR 1.0, 95% CI 1.0–1.0), the increased DNA methylation signal from the biomarker set was significantly associated with worse overall survival in cancer patients (HR 1.29, 95% CI 1.08–1.55, *p* = 0.006). The Kaplan–Meier plots (Additional file 1: Figure S3) show that the cancer patients with lower biomarker DNA methylation signal have better overall survival, while there was no difference in survival between the high and low CA 19-9 groups (Additional file 1: Figure S3). Multivariate analysis including several clinical factors (Additional file 1: Table S2) confirmed these findings. In summary, these results demonstrate that our liquid biopsy assay, using our biomarker set, can distinguish between benign and malignant pancreatic disease with very high sensitivity and specificity.

**Fig. 2:**
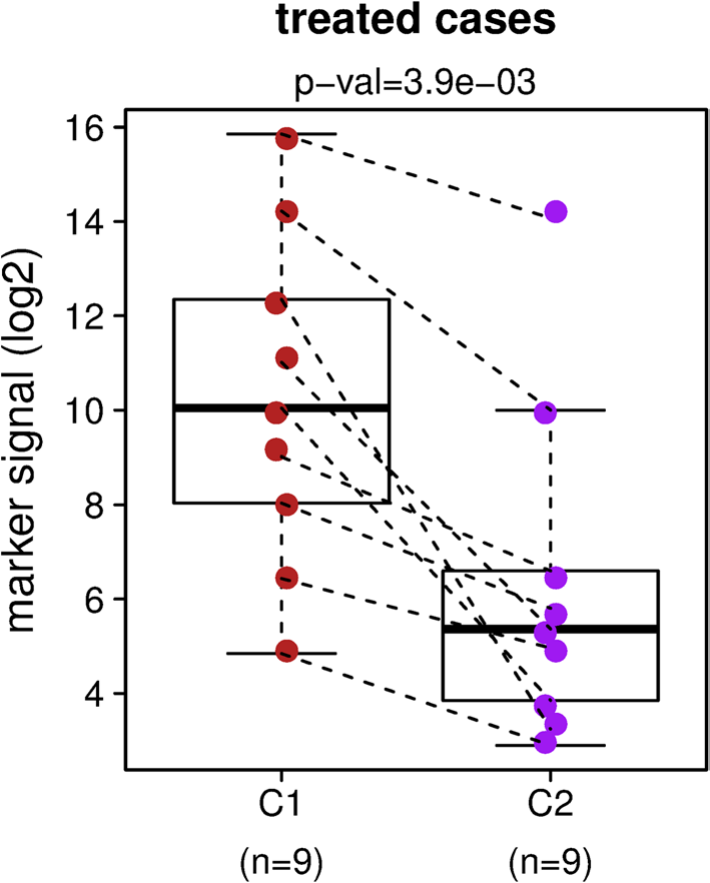
The DNA methylation biomarker set detects difference in the disease burden after treatment of pancreatic adenocarcinoma. The boxplots show the mean DNA methylation signal per marker for 9 pairs of blood samples taken before the treatment and 4 weeks after the first cycle of treatment started. The y-axis is in a log2 scale. *p* = 3.9 × 10^–3^ by Wilcoxon signed rank test

To evaluate the utility of the biomarkers for disease monitoring, we followed the changes in DNA methylation signal from the biomarker set longitudinally during pancreatic cancer treatment. The treatment consisted of an aggressive clinical trial regimen using five drugs cocktail (NAPPCG): nivolumab, albumin-bound paclitaxel, paricalcitol, cisplatin, and gemcitabine. The DNA methylation signal of the biomarkers was analyzed in 9 pairs of plasma samples taken before the treatment started (C1) and 4 weeks after the first cycle of treatment (C2). Figure 2 shows a statistically significant decrease (*p* value = 3.9 × 10^–3^) in the biomarker signal across all treated subjects. The decrease in the median biomarker signal within the first cycle of the treatment was by a factor of 25.6-fold (Fig. 2). The detected decrease in the biomarker DNA methylation may correlate with a lower disease burden resulting in decline of the tumor-derived DNA in plasma as a response to pancreatic cancer treatment. In summary, this suggests that the biomarker set could be potentially useful for monitoring patients undergoing pancreatic cancer treatment.

## Discussion

Pancreatic cancer is a highly lethal malignancy with poor overall survival due to silent progression of disease prior to developing clinical symptoms. Even in the minority of patients who are diagnosed at an early stage and are candidates for curative-intent surgery, postoperative recurrence after surgical resection is very frequent. Due to these findings, efforts to identify minimally invasive ways to provide earlier diagnosis and enhanced prognostication are increasingly warranted. Liquid biopsies, using the cell-free DNA fraction of blood samples could be a promising tool to accomplish this goal.

The results from the present study indicate that our biomarker set can reliably distinguish metastatic pancreatic cancer from benign pancreatic cysts with high sensitivity and specificity. In addition, despite the relatively small number of cases analyzed and a limited follow-up time, an increased biomarker signal had a significant association with overall worse survival (HR = 1.29, *p* = 0.006). The reason for increased biomarker set DNA methylation signal in more severe cases is likely the overall higher mass of tumor cells in the body resulting in more ctDNA in blood. Carbohydrate antigen 19-9 (CA 19-9) is one of the established biomarkers for pancreatic cancer diagnostics and prognostication [25]. Surprisingly we did not observe any predictive value of CA 19-9 (HR = 1.0) in our limited cohort of 19 malignant cases. This indicates that the presented biomarker set has a certain prognostic value, potentially better than CA 19-9. Furthermore, the biomarker set detected differences in the disease burden after treatment of pancreatic adenocarcinoma. These observations agree with our previous study in non-small cell lung cancer patients [22].

There are several studies on cell-free DNA methylation analysis using plasma or serum as an analyte to diagnose or monitor pancreatic cancer (reviewed in [26]). In one study, methylation-specific PCR targeting 8 loci of blood-derived DNA in 95 patients with PDAC yielded only moderate accuracy with a sensitivity of 76% at 83% specificity [27]. The loci used in their broader panel were selected based on a literature search for previously published biomarkers, which is unlike our unbiased bioinformatics approach that yielded highly specific and sensitive biomarkers [20]. In another study, a two loci liquid biopsy test exhibited 97.3% sensitivity at 91.6% specificity, AUC 0.95, for PDAC detection [28]. Another previous study identified 13 methylation marker set that could detect pancreatic cancer with 92% specificity at 97.5% specificity [29]. In comparison, the performance of our liquid biopsy assay on our limited cohort of pancreatic disease patients presented here was 100% sensitivity at 95% specificity to distinguish between malignant and benign disease. Collectively, these studies support further development of liquid biopsy assays for multiple clinical applications in PDAC management. We are currently validating our findings in a prospective case–control study for the detection of early-stage localized PDAC and a longitudinal observational study determining whether the biomarker set can detect minimal residual disease in PDAC in the neoadjuvant therapy setting. This minimally invasive blood-based assay using DNA methylation biomarker panel could be a promising tool for detection of early PDAC disease and assist in the management of pancreatic cancer patients.

## Supplementary Information

**Additional file 1.** Supplementary Information for Liquid biopsy, using a novel DNA methylation signature, distinguishes pancreatic adenocarcinoma from benign pancreatic disease.

## Data Availability

Not applicable.

## Abbreviations

AUC: Area under the curve
ROC: Receiver operating characteristic
cfDNA: Cell-free DNA
CA 19-9: Carbohydrate antigen 19–9
NAPPCG: Nivolumab, albumin-bound paclitaxel, paricalcitol, cisplatin, and gemcitabine
qRT-PCR: Quantitative real-time PCR
PDAC: Pancreatic adenocarcinoma
NSCLC: Non-small cell lung cancer
