# Supplementary material for: Liquid biopsy, using a novel DNA methylation signature, distinguishes pancreatic adenocarcinoma from benign pancreatic disease
Source: Clin Epigenetics. 2022 Feb 22;14:28. doi: 10.1186/s13148-022-01246-2 (PMC8864826; doi:10.1186/s13148-022-01246-2)
Supplement: Supplementary file 1 — Additional file 1. Supplementary Information for Liquid biopsy, using a novel DNA methylation signature, distinguishes pancreatic adenocarcinoma from benign pancreatic disease. [file 13148_2022_1246_MOESM1_ESM.pdf]

Liquid biopsy, using a novel DNA methylation signature, distinguishes pancreatic adenocarcinoma from benign pancreatic disease

Vrba et al.

Supplemental materials:

Figure S1 ……………………… 2

Figure S2 ……………………… 3

Figure S3 ……………………… 4

Table S1 ……………………… 5

Table S2 ……………………… 6

Supplementary references 7

Figure S1

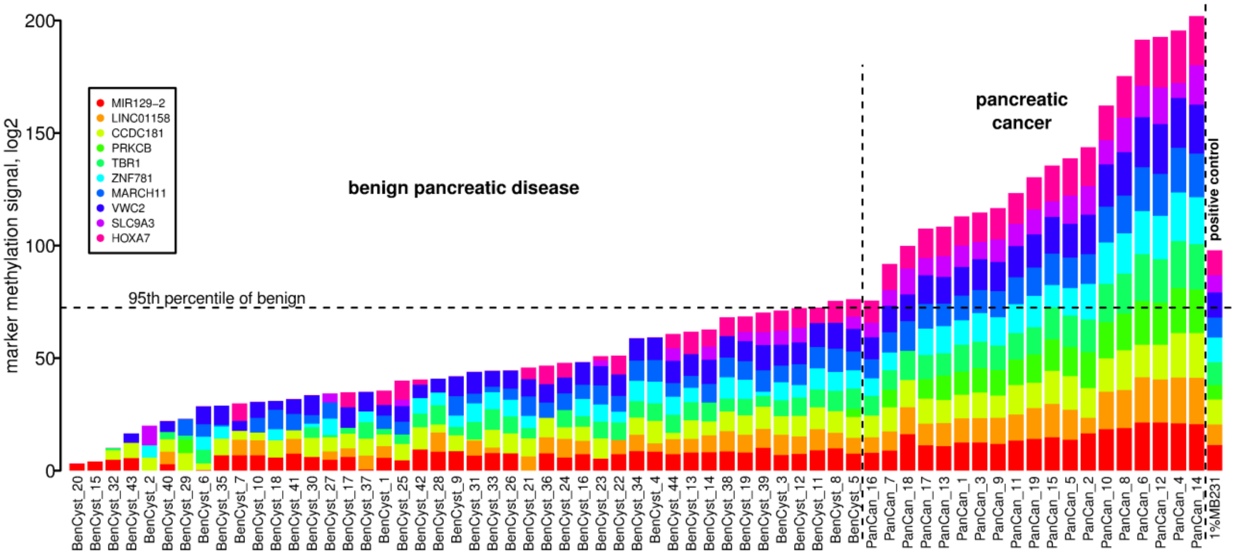

DNA methylation signal from the biomarker set on a cohort of 44 control pancreatic cysts subjects (left part) and 19 pancreatic adenocarcinoma cases (right portion). The 95th percentile of the cumulative DNA methylation of the pancreatic cyst cohort is represented by the horizontal dashed line. The y axis is in a log2 scale.

Figure S2

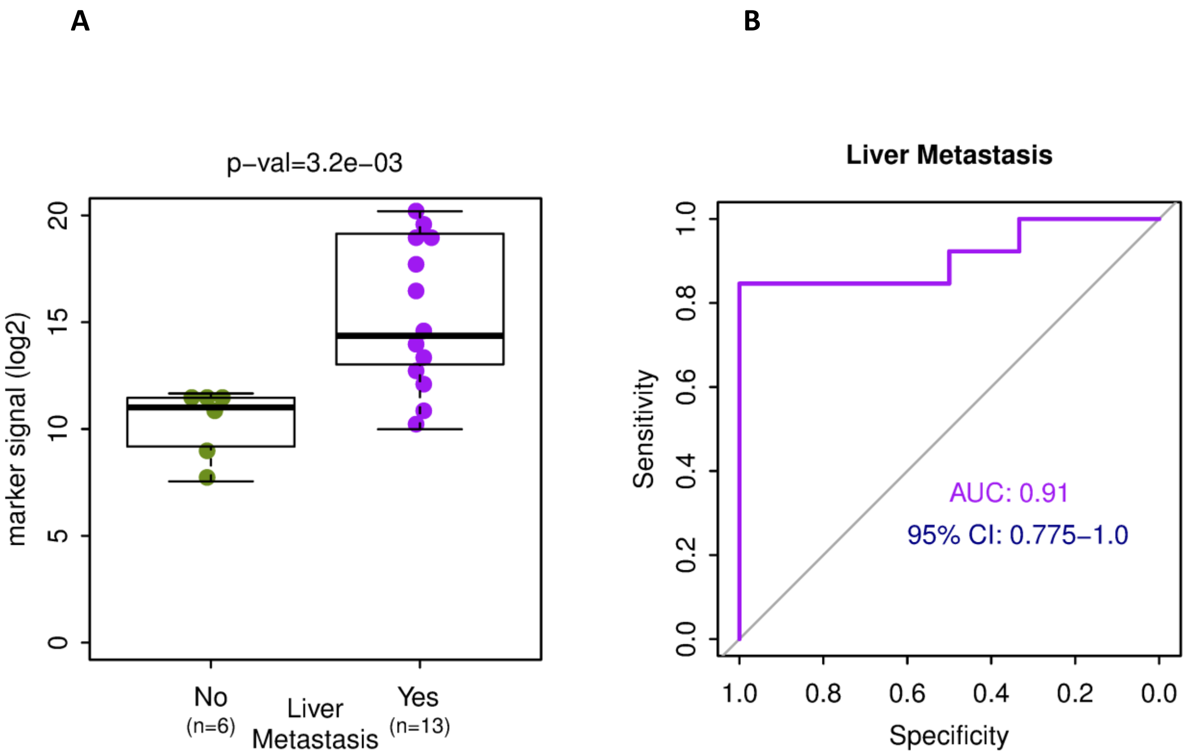

The DNA methylation biomarker set signal is increased in PDAC patients with liver metastases. All the cases were diagnosed with Stage IV disease. A) The boxplots show the mean DNA methylation signal per marker for pancreatic adenocarcinoma cases with no liver metastasis (n= 6) and with liver metastasis (n = 13). The y axis is in a log2 scale. P = 3.2 x10^{-3} by Wilcoxon rank sum test. B) The receiver operating characteristic (ROC) analysis of the biomarker set signal from cases without liver metastasis and cases with liver metastasis. AUC – area under the curve, CI – confidence interval.

Figure S3

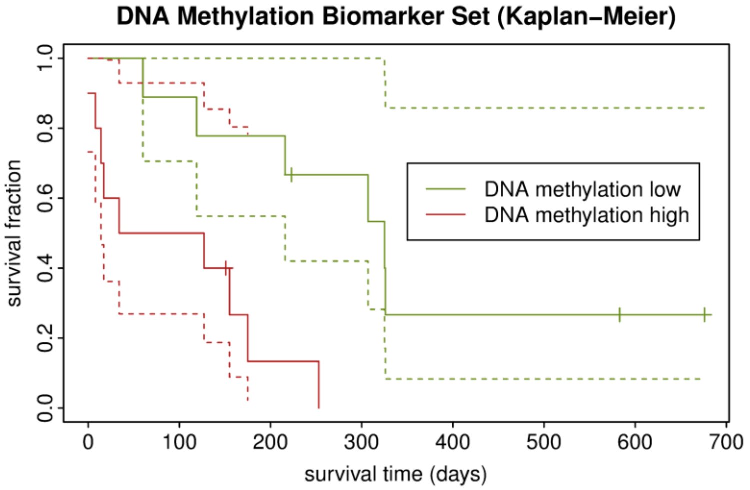

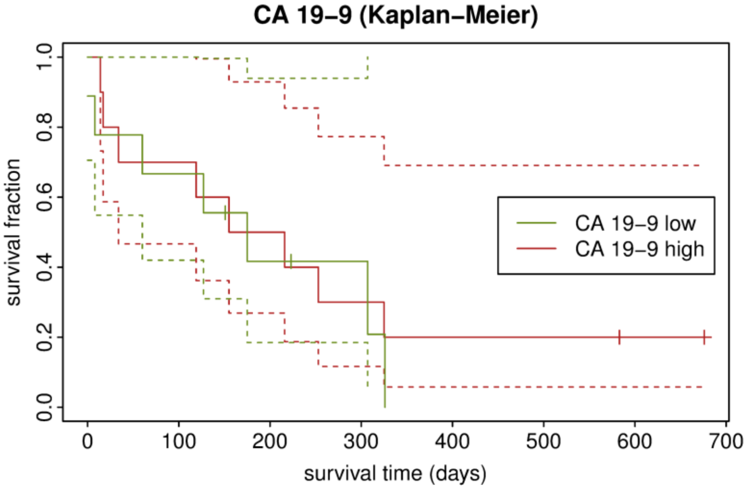

Kaplan-Meier plots for 19 pancreatic cancer cases. Top - DNA methylation signal from the biomarkers as predictor. The low group (n=9, signal range 7.5-12.3), the high group (n=10, signal range 13.0 -20.2). Bottom - CA 19-9 as predictor. The low group (n=9, range 3-1000), the high group (n=10, range 1173 - 56534). 95% confidence bounds are indicated.

Table S1

Biological roles of genes in DNA methylation signature

CpG.ID	Gene	Function
cg14416371	MIR129-2	Cell proliferation and apoptosis<br>Metastasis inhibitor<br>Reverses EMT [1]
cg08189989	LINC01158	Cell proliferation, migration, and invasion regulator<br>Angiogenesis<br>TGFB signaling[2]
cg00100121	CCDC181	Generic transcription and gene expression regulator[3]
cg03306374	PRKCB	Regulator of proliferation, apoptosis, migration, and differentiation[4]
cg01419831	TBR1	Transcription factor involved in several developmental pathways[5]
cg25875213	ZNF781	Transcription regulator[6]
cg00339556	MARCH11	Vesicle trafficking and protein degradation regulator[7]
cg01893212	VWC2	TGFb/BMP signaling regulator involved in cell adhesion[8]
cg14732324	SLC9A3	Solute carrier involved in pH regulation[9]
cg07302069	HOXA7	Transcription factor that regulates gene expression, morphogenesis, and differentiation[10]

Table S2

Multivariate analysis of survival

Parameter	HR	lower .95	upper .95	z	Pr(>|z|)
Marker Methylation Signal	1.681	1.158	2.439	2.731	0.00632
CA 19-9	1.000	1.000	1.000	0.334	0.73865
Tumor Burden (cm) [a]	1.232	0.967	1.569	1.687	0.09154
Number of Metastatic Sites	0.075	0.012	0.475	-2.747	0.00602
Liver Metastases (Y/N)	1.261	0.196	8.098	0.245	0.80672
Number of Lymph Nodes	2.694	1.324	5.479	2.735	0.00625
Treatment (Y/N) [b]	0.173	0.022	1.347	-1.675	0.09386
Surgery Prior (Y/N) [c]	6.906	0.677	70.431	1.631	0.10289

a - the sum of the longest diameter of the solid tumor lesions + the sum of the short axis of any LNs (cm)

b - thirteen patients were treated after the metastatic cancer diagnosis and methylation analysis

c - four patients, where the metastatic disease was recurrent, underwent surgery in the past, prior to metastatic diagnosis and methylation analysis

Supplementary references

1. Gao Y, Feng B, Han S, et al. MicroRNA-129 in Human Cancers: from Tumorigenesis to Clinical Treatment. Cell Physiol Biochem 2016;39:2186-202.
2. Seles M, Hutterer GC, Fosselteder J, et al. Long Non-Coding RNA PANTR1 is Associated with Poor Prognosis and Influences Angiogenesis and Apoptosis in Clear-Cell Renal Cell Cancer. Cancers (Basel) 2020;12.
3. Schwarz T, Prieler B, Schmid JA, Grzmil P, Neesen J. Ccdc181 is a microtubule-binding protein that interacts with Hook1 in haploid male germ cells and localizes to the sperm tail and motile cilia. European Journal of Cell Biology 2017;96:276-88.
4. Patergnani S, Marchi S, Rimessi A, et al. PRKCB/protein kinase C, beta and the mitochondrial axis as key regulators of autophagy. Autophagy 2013;9:1367-85.
5. Mihalas AB, Hevner RF. Control of Neuronal Development by T-Box Genes in the Brain. Curr Top Dev Biol 2017;122:279-312.
6. Mungala N, Hammand, H., Vrba, L., Oshiro, M., Wertheim, B., Roe, D., Gavini, H., Latura, L., Pennington, D., Dalgai, S., Babiker, H.M., Elquza, E., Scott, A.J., Nelson, M., Futscher, B.W., and Shroff, R.T. . Detection of pancreatic cancer using a novel blood-based DNA methylation signature. Journal of Clinical Oncology 2020 38.
7. Morokuma Y, Nakamura N, Kato A, et al. MARCH-XI, a novel transmembrane ubiquitin ligase implicated in ubiquitin-dependent protein sorting in developing spermatids. J Biol Chem 2007;282:24806-15.
8. Almehmadi A, Ohyama Y, Kaku M, et al. VWC2 Increases Bone Formation Through Inhibiting Activin Signaling. Calcif Tissue Int 2018;103:663-74.
9. Xu H, Ghishan FK, Kiela PR. SLC9 Gene Family: Function, Expression, and Regulation. Compr Physiol 2018;8:555-83.
10. Gehring WJ, Hiromi Y. Homeotic genes and the homeobox. Annu Rev Genet 1986;20:147-73.
